# Identification of a highly conserved valine-glycine-phenylalanine amino acid triplet required for HIV-1 Nef function

**DOI:** 10.1186/1742-4690-9-34

**Published:** 2012-04-27

**Authors:** Pieter J Meuwissen, Bettina Stolp, Veronica Iannucci, Jolien Vermeire, Evelien Naessens, Kalle Saksela, Matthias Geyer, Guido Vanham, Kevin K Arien, Oliver T Fackler, Bruno Verhasselt

**Affiliations:** 1 Department of Clinical Chemistry, Microbiology, and Immunology, Ghent University, Ghent, (B-9000), Belgium; 2 Department of Infectious Diseases, Virology, University Hospital Heidelberg, INF 324, Heidelberg, (D-69120), Germany; 3 Department of Virology, Haartman Institute, University of Helsinki and Helsinki University Central Hospital, Helsinki, (FIN-00014), Finland; 4 Max Planck Institute for Molecular Physiology, Dortmund, (D-44227), Germany; 5 Department of Biomedical Sciences, Virology Unit, Institute of Tropical Medicine, Antwerp, (B-2000), Belgium; 6 Present Address: Department of Biomedical Sciences , Virology Unit, Institute of Tropical Medicine, Antwerp, Belgium

**Keywords:** HIV, Nef, Sequence motifs, SH3 domain binding, Cytoskeleton, Lck, Receptor downregulation, Infectivity, Replication

## Abstract

**Background:**

The Nef protein of HIV facilitates virus replication and disease progression in infected patients. This role as pathogenesis factor depends on several genetically separable Nef functions that are mediated by interactions of highly conserved protein-protein interaction motifs with different host cell proteins. By studying the functionality of a series of *nef* alleles from clinical isolates, we identified a dysfunctional HIV group O Nef in which a highly conserved valine-glycine-phenylalanine (VGF) region, which links a preceding acidic cluster with the following proline-rich motif into an amphipathic surface was deleted. In this study, we aimed to study the functional importance of this VGF region.

**Results:**

The dysfunctional HIV group O8 *nef* allele was restored to the consensus sequence, and mutants of canonical (NL4.3, NA-7, SF2) and non-canonical (B2 and C1422) HIV-1 group M *nef* alleles were generated in which the amino acids of the VGF region were changed into alanines (VGF→AAA) and tested for their capacity to interfere with surface receptor trafficking, signal transduction and enhancement of viral replication and infectivity. We found the VGF motif, and each individual amino acid of this motif, to be critical for downregulation of MHC-I and CXCR4. Moreover, Nef’s association with the cellular p21-activated kinase 2 (PAK2), the resulting deregulation of cofilin and inhibition of host cell actin remodeling, and targeting of Lck kinase to the trans-golgi-network (TGN) were affected as well. Of particular interest, VGF integrity was essential for Nef-mediated enhancement of HIV virion infectivity and HIV replication in peripheral blood lymphocytes. For targeting of Lck kinase to the TGN and viral infectivity, especially the phenylalanine of the triplet was essential. At the molecular level, the VGF motif was required for the physical interaction of the adjacent proline-rich motif with Hck.

**Conclusion:**

Based on these findings, we propose that this highly conserved three amino acid VGF motif together with the acidic cluster and the proline-rich motif form a previously unrecognized amphipathic surface on Nef. This surface appears to be essential for the majority of Nef functions and thus represents a prime target for the pharmacological inhibition of Nef.

## Background

Primate immunodeficiency virus Nef is a 25–35 kDa protein expressed by a conserved open reading frame, which partially overlaps with the 3 long terminal repeat (LTR) in the genome of HIV-1, HIV-2 or simian immunodeficiency virus (SIV). Nef is expressed early in the viral life cycle and is required for efficient viral replication and disease progression in the infected host. Therefore, the absence of Nef slows down or completely abolishes the progression towards acquired immunodeficiency syndrome (AIDS) [1,2]. Interestingly, a strong selective pressure has been demonstrated for a functional *nef* gene, since macaques infected with non-pathogenic, SIV *nef*-mutant show *in vivo* repair of the reading frame and subsequent progression to AIDS-like disease [3]. Moreover, isolated expression of Nef in transgenic mice induces a strong depletion of CD4^+^ cells, resembling an AIDS-like phenotype [4,5]. Additionally, cohorts of patients infected with HIV variants harboring Nef mutations and/or deletions in *nef* show a delayed onset of AIDS. While these studies clearly established Nef as a critical factor for AIDS pathogenesis, the underlying molecular mechanism remains to be fully elucidated.

Nef associates with host cell membranes through the N-terminal myristoyl group and functions as an adaptor protein, promoting viral pathogenicity probably by interacting with several classes of host cell proteins, mainly protein kinases and components of the endocytic trafficking machinery. For instance, Nef reduces surface expression of the HIV entry receptor CD4 and co-receptors CCR5 and CXCR4 to prevent superinfection of already productively infected cells and possibly aiding virion release from these cells [5-9]. Nef also leads to reduced cell surface expression of MHC class I and MHC class II molecules to facilitate immune evasion of infected cells [6-8]. Finally, Nef interferes with the T cell receptor signal transduction machinery and enhances virion infectivity and viral replication [9,10].

Mutational analysis revealed that individual actions of Nef have distinct structural correlates. The protein has a flexible, myristoylated N-terminal anchor domain of variable length followed by a loop section containing a proline-rich type II helix, a core domain and a C-terminal flexible loop containing an endocytic di-leucine based sorting motif that is required for most trafficking functions of Nef [11]. Specifically, downregulation of CD4 requires conserved amino acid residues located at the N-terminal arm and the disordered C-terminal loop of Nef, whereas downregulation of MHC-I and CXCR4 depend on a cluster of acidic amino acid residues (EEEE, stretch of glutamic or aspartic acids) as well as the neighboring proline-rich motif that is part of a Src homology domain 3 (SH3) binding surface of Nef (PxxPxVPxRP, first four amino acids represented by PxxP, x is an unspecified amino acid). A large number of cellular partners have been identified and for some of them the binding sites on Nef have been mapped [11,12].

In this study, we analyzed a panel of HIV-1 and HIV-2 *nef* alleles derived from clinical HIV isolates and identified a *nef* allele that was naturally mutated in an amphipathic stretch of amino acids in the PxxP loop region, compromising both the acidic cluster and the proline-rich motif. Analysis of this and other Nef mutants revealed a crucial role of the three amino acid valine-glycine-phenylalanine (VGF) motif which links the acidic cluster to the proline-rich motif. Without affecting the stability of the protein, specific mutations of the VGF motif interfered with a wide variety of Nef functions which are known to depend on the integrity of the proline-rich motif, including MHC-I and CXCR4 downregulation, association of PAK2 and Hck kinases and interference with T cell receptor signaling as well as enhancement of viral infectivity and replication. For surface receptor trafficking and cytoskeletal functions each individual amino acid of the triplet is important; while for targeting of Lck kinase to the trans-golgi-network and viral infectivity, especially the phenylalanine of the triplet was essential. We propose that the highly conserved three amino acid VGF motif together with the adjacent acidic cluster and proline-rich motif form a previously unrecognized amphipathic surface on Nef that is essential for the majority of Nef functions.

## Results

### *Nef* alleles from different clades of HIV isolates modulate surface expression levels of human receptors

**Figure 1:**
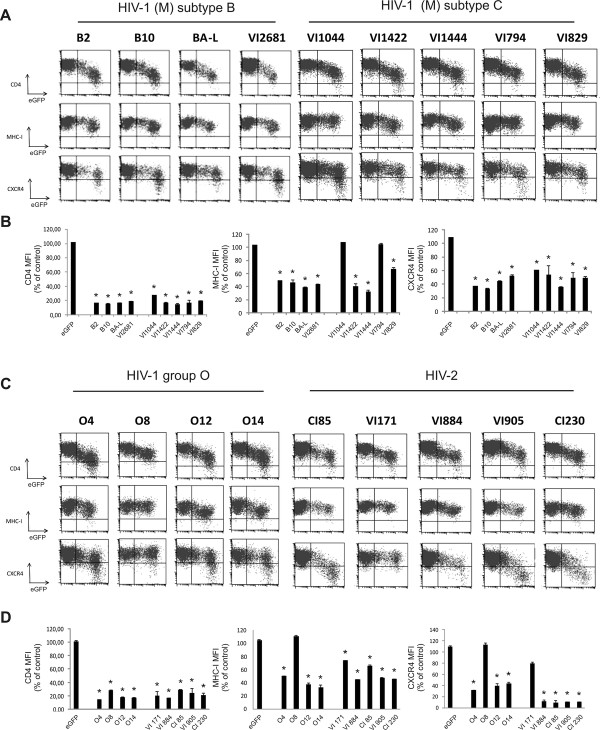
** Effect of primary HIV-1 and HIV-2 Nef proteins on surface receptor expression**. Jurkat CD4-CCR5 cells were transduced with retroviral vectors expressing the indicated *nef* alleles and eGFP (Nef-IRES-eGFP) and were analyzed by flow cytometry. (A and C) Bivariate dot plots show expression of CD4, MHC-I, CXCR4 as a function of eGFP (Nef) expression. Dot plots shown are representative of results obtained in at least 3 independent experiments. (B and D) To determine relative CD4, MHC-I and CXCR4 surface levels, the MFI of transduced cells (eGFP^+^) was divided by the MFI of untransduced cells (eGFP^-^) in the same culture. Data represent the mean relative expression and standard deviations from at least 3 independent experiments. eGFP represents the value for control transduced cells expressing only the marker gene but no Nef. An asterisk (*) over a bar indicates a statistically significant difference between the experimental construct compared with the eGFP control (P < 0.01).

Nef downregulates the expression of cell surface markers, such as CD4, MHC-I and CXCR4. We initially set out to extend available data on the conservation of these functions [13-15] in a series of clinical HIV-1 (NIH AIDS Reference and Reagent Program) and HIV-2 isolates (Institute for Tropical Medicine, Antwerp, Belgium) that we used before to assess viral fitness hierarchy [16]. *Nef* alleles were sequenced ( Additional file 1: Figure S1) and amplified from infected PBMCs and expressed by retroviral transduction in Jurkat CD4-CCR5 cells. Transduced cells were subsequently analysed by flow cytometry for surface receptor levels. Transduced cells express both Nef and an eGFP marker protein at correlating levels [17]. As a control, we included a virus encoding for eGFP only. In order to quantify the extent of receptor downregulation, the ratio between the mean fluorescence intensity (MFI) of transduced cells (eGFP^+^) was divided by the MFI of untransduced cells in the same culture. Expression of all *nef* alleles resulted in at least 5-fold decrease of CD4 surface expression, confirming the functionality of these Nef proteins. However, the effect of the isolated *nef* alleles on MHC-I and CXCR4 surface expression was more variable (Figure 1). The majority of the isolated HIV-1 group M, HIV-1 group O and HIV-2 Nef proteins downmodulated the expression of MHC-I and CXCR4 resulting in a 2- to 3-fold reduction in surface expression. Interestingly, two HIV-1 subtype C alleles (VI1044 and VI794; Figure 1 and 1B) and one HIV-1 group O allele (O8) did not show any effect on MHC-I downregulation (Figure 1C and 1D). All Nef proteins, except Nef O8 and HIV-2 VI171, downregulated the surface expression of CXCR4 (Figure 1C and 1D). In line with previous reports [15], *nef* alleles isolated from HIV-2 strains were much more efficient in downregulating CXCR4 than HIV-1 *nef* alleles (near 10-fold reduction of CXCR4 surface expression for HIV-2 compared to 2-3-fold for HIV-1). Clearly, there was no correlation between the ability of various Nef proteins to downregulate CXCR4, MHC-I and CD4, confirming that the mechanisms of CD4 and MHC-I downregulations are at least in part genetically separable [15,18,19] and also suggesting that downregulation of CXCR4 and MHC-I depend on the interaction of Nef with distinct cellular ligands and machineries.

### Mutational analysis of the natural Nef mutant HIV-1 O8

**Figure 2:**
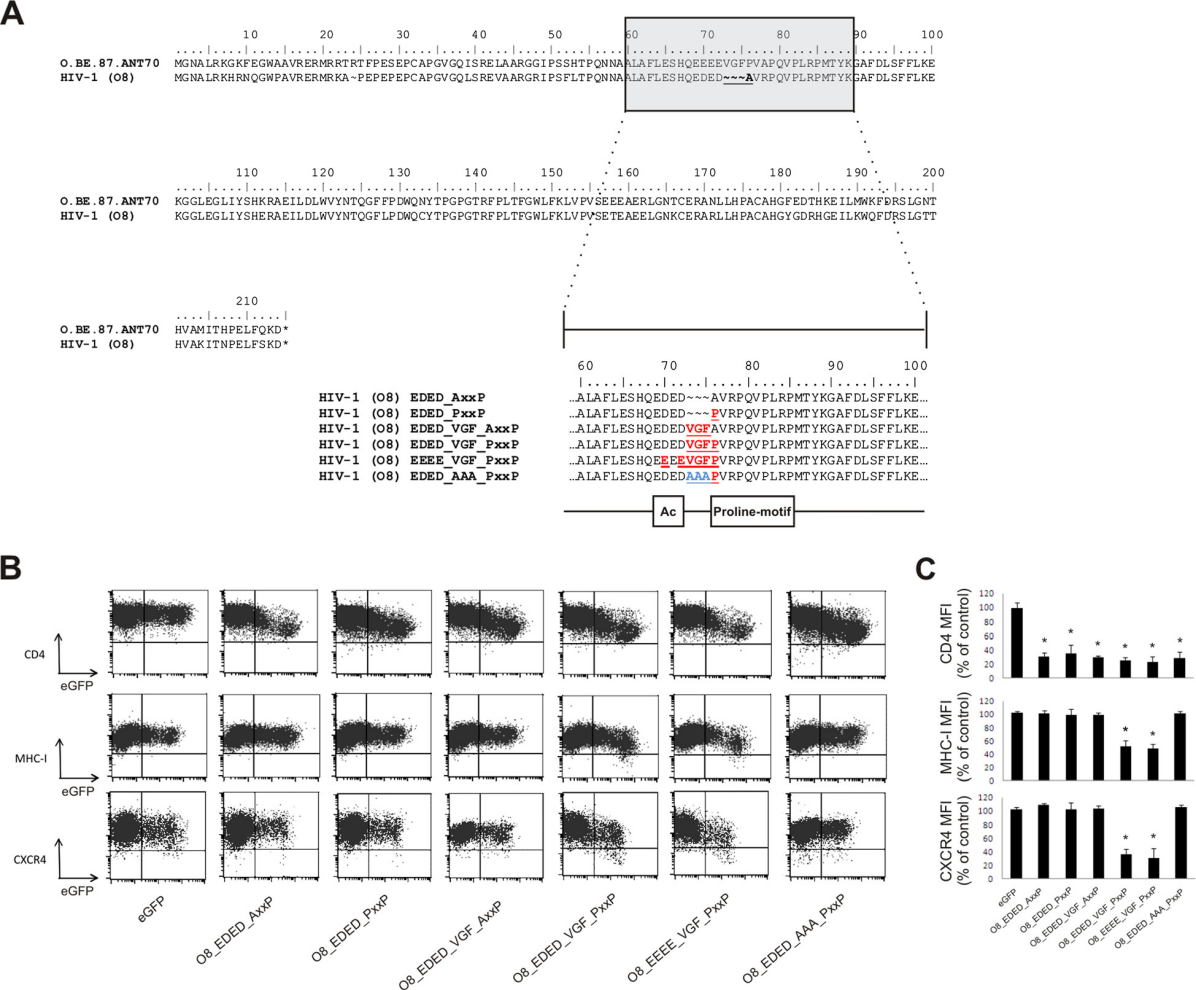
** Mutational analysis of Nef O8 identifies functional importance of the VGF region which links the acidic cluster and the proline-rich motif.** (A) Alignment of the sequence of Nef O8 relative to a subtype O reference allele (O.BE.87.ANT70) obtained from the Los Alamos HIV sequence database reveals that the VGF motif is deleted in Nef O8. In addition, the first proline of the PxxP motif changed into alanine. A panel of mutants was generated in which the different mutations were sequentially reverted to their consensus variant (marked in red) or to a variant in which VGF was changed into triple alanines (AAA; blue). As reference, boxes below reverted sequences mark the acidic cluster (Ac) and proline-rich motif (Proline-motif) (B) Jurkat CD4-CCR5 cells were transduced with retroviral vectors expressing either eGFP alone or together (Nef-IRES-eGFP) with the indicated *nef* mutant and were analyzed by flow cytometry. Bivariate dot plots show expression of CD4, MHC-I, CXCR4 as a function of eGFP (Nef) expression. Dot plots shown are representative for results obtained in 3 independent experiments. (C) To determine relative surface levels, the MFI of transduced cells (eGFP^+^) was divided by the MFI of untransduced cells (eGFP^-^) in the same culture. Data represent the mean relative expression and standard deviations from 3 independent experiments. * over a bar indicates a statistically significant difference between the experimental construct compared with the eGFP control (P < 0.01).

In our panel of primary *nef* alleles, we identified 4 *nef* alleles that were deficient for MHC-I and/or CXCR4 downregulation. A subtype C *nef* allele (VI1044) contained an insertion of a few amino-acids, which interrupted the structure of the N-proximal α-helical region, explaining the lack of MHC-I downregulation capacity of this allele ( Additional file 1: Figure S1C) [20]. *Nef* alleles VI794 (subtype C) and VI171 (HIV-2) did not show any mutations in regions known to be important for Nef functions ( Additional file 1: Figure S1C and Figure S1D). By contrast, Nef O8 showed mutations in an amphipathic stretch of amino acids in the Nef core domain encompassing the acidic cluster and the proline-rich motif (Figure 2A). More specifically, a point mutation changed the first proline of the polyproline stretch into an alanine. In addition to this point mutation in the PxxP motif, Nef O8 also showed a deletion of a three peptidic motif, i.e. valine-glycine-phenylalanine (VGF), located right in between the acidic cluster and the polyproline stretch. Based on the sequences found in the Los Alamos HIV sequence database consulted in December 2011, the VGF was highly conserved in HIV-1 and SIVcpz (V: 99.20 %; G: 98.85 %; F: 99.52 %). [21,22]. HIV-2 *nef* alleles instead contain a highly conserved VGV motif (V: 93.06 %; G: 97.22 %; V: 79.17 %, [22]) indicating that these residues are important for optimal Nef function. Despite intensive research on the functions of conserved regions in Nef, the importance of the VGF/V region has not yet been appreciated or investigated thoroughly.

To evaluate the individual contribution of the observed mutations for the defective phenotype of the O8 *nef* allele, we generated a panel of mutants in which the different mutations were sequentially reverted to their consensus variant (Figure 2A) and re‐evaluated their ability to downregulate surface molecules. Neither restoration of the AxxP sequence into PxxP (*i.e.* EDED_PxxP) nor introduction of the deleted amino acids (*i.e.* EDED_VGF_AxxP) restored the capacity to interfere with MHC-I and CXCR4 surface expression (Figure 2B and 2C). Only simultaneous restoration of the PxxP and the VGF motif into the consensus variant (*i.e.* EDED_VGF_PxxP or EEEE_VGF_PxxP) rescued CXCR4 and MHC-I downregulation by O8 Nef. Interestingly, when all three amino acids of the VGF motif in Nef O8 were converted into alanine (*i.e.* EDED_AAA_PxxP), in the presence of an intact polyproline stretch, the capacity to affect MHC-I and CXCR4 surface expression was again lost (Figure 2B and 2C), indicating that specific amino acids in both motifs are important.

### An amphipathic surface in Nef is important for downregulation of CXCR4 and MHC

**Figure 3:**
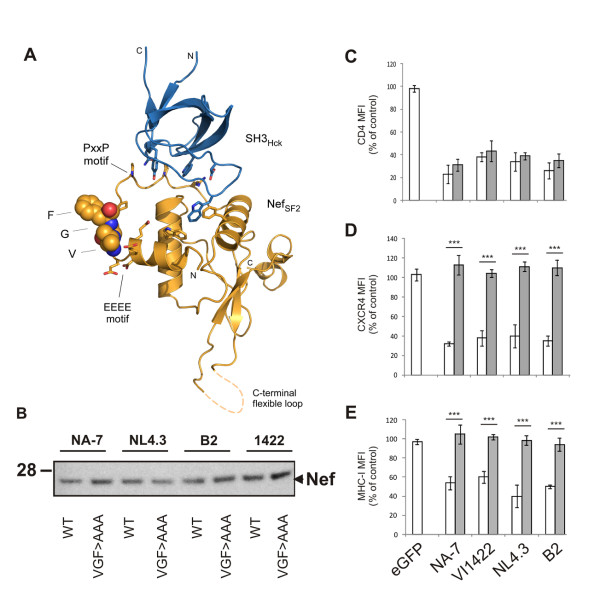
** The VGF linker region is critical for downregulation of CXCR4 and MHC-I by HIV-1 Nef.** To evaluate the functional importance, VGF→AAA mutants of several Nef proteins were expressed and evaluated for their effect on cell surface receptor expression. (A) Display of the VGF motif in the structure of Nef_SF2_ (yellow ribbon) bound to an Hck-SH3 domain (blue ribbon). VGF residues, which are deleted in Nef allele O8, are indicated as space-filling model. Residues of the preceding acidic cluster and the following PxxP motif are shown as stick representation. The structure is based on PBD accession number 3REA [23]. (B) WT or VGF→AAA Nef-IRES-eGFP transduced Jurkat CD4-CCR5 cells were sorted, lysed and analyzed for Nef expression. The figure shows a Western blot of whole cell lysates stained with sheep-anti-Nef antiserum. The marker line indicates position of a 28 kDa standard. Loading control staining (actin) was similar over all samples (not shown). (C-E) Relative CD4, MHC-I, CXCR4 surface levels measured by flow cytometry on Jurkat CD4-CCR5 cells transduced with retroviral vectors expressing either eGFP alone or together with the indicated *nef* alleles and mutants. To determine relative surface levels, the MFI of transduced cells (eGFP^+^) was divided by the MFI of untransduced cells (eGFP^-^) in the same culture. Data represent the mean relative expression and standard deviations obtained from 3 independent experiments, wild-type allele (open columns) next to the corresponding VGF→AAA mutants (grey shaded columns). *** over a bar indicates a statistically significant difference between the VGF→AAA mutant compared to the wild-type allele (P < 0.001).

Depiction of the VGF motif within the structure of Nef showed that this motif is located within a loop region of Nef directly preceding the PxxP motif (Figure 3A). The motif is at the surface of Nef and highly accessible for protein-protein interactions [23]. To prove the functional conservation of the VGF motif, we initially evaluated the importance of this domain for MHC-I and CXCR4 downregulation in *nef* alleles derived from HIV-1 group M, two canonical group (M) subtype B strains (i.e. NL4.3 and NA-7) and two non-canonical isolates: subtype B (i.e. B2) and subtype C (i.e. VI1422). Mutations were designed to generate triple mutations of VGF into alanines (VGF→AAA).

To exclude the possibility that the introduced mutation could affect the stability of the Nef protein, we transduced Jurkat T cells to express wild type (WT) Nef proteins and their mutant counterparts and evaluated their expression by Western blotting (Figure 3B). Equal amounts of Nef protein were present in sorted cell lysates, indicating that the VGF to AAA mutation did not affect the stability of the protein. Next, we evaluated the influence of these mutant Nef proteins on surface receptor expression. Analysis of the surface expression of CD4, CXCR4 and MHC-I by flow cytometry did not reveal a deleterious effect of VGF→AAA mutation on CD4 downregulation of all *nef* alleles analyzed, indicating that the expressed Nef mutants retained this function (Figure 3C), as did O8 Nef. However, these mutants were completely defective for MHC-I and CXCR4 downregulation (Figure 3D and 3E). This finding indicates that the VGF motif is not only important for downregulation of these surface molecules in Nef O8, but also in HIV-1 group M Nef proteins.

**Figure 4:**
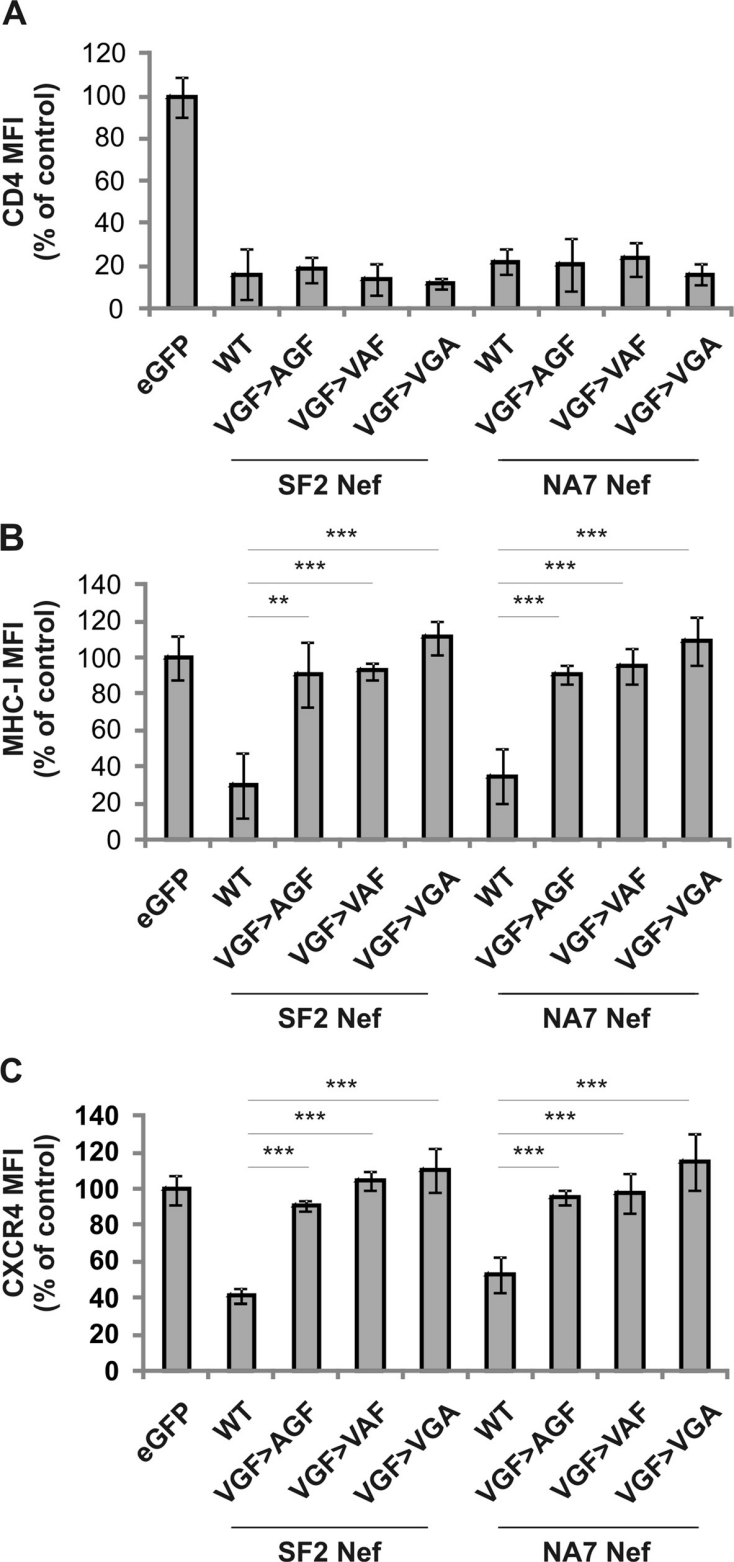
** Sequence specific requirement of V-G-F for Nef function.** Panels A to C show relative surface levels of CD4, MHC-I, CXCR4 respectively, measured by flow cytometry on Jurkat CD4-CCR5 cells transduced with retroviral vectors expressing either eGFP alone or together with the indicated *nef* alleles and mutants. To determine relative surface levels, the MFI of transduced cells (eGFP^+^) was divided by the MFI of untransduced cells (eGFP^-^) in the same culture. Data represent the mean relative expression and standard deviations obtained from 3 independent experiments,** over a bar indicates P < 0.01 and *** indicates P < 0.001.

Each amino acid residue of the VGF motif is highly conserved, suggesting a sequence specific requirement. However, the triple mutation of the VGF stretch might be structurally drastic and alter the functionality of adjacent motifs. To exclude this and to confirm the sequence specific requirement of the VGF motif, a panel of single mutants of the VGF motif, in which the different amino acids are changed into alanine (AGF, VAF and VGA), was generated and evaluated for their effect on Nef function. Similarly to the VGF→AAA mutant, single mutants failed to down regulate MHC-I and CXCR4, while maintaining the capacity to downregulate CD4 (Figure 4). These results indicate that the VGF stretch as such is required for downregulation of MHC-I and CXCR4.

### Integrity of the VGF motif is essential for effects of Nef on actin dynamics and Lck localization

**Figure 5:**
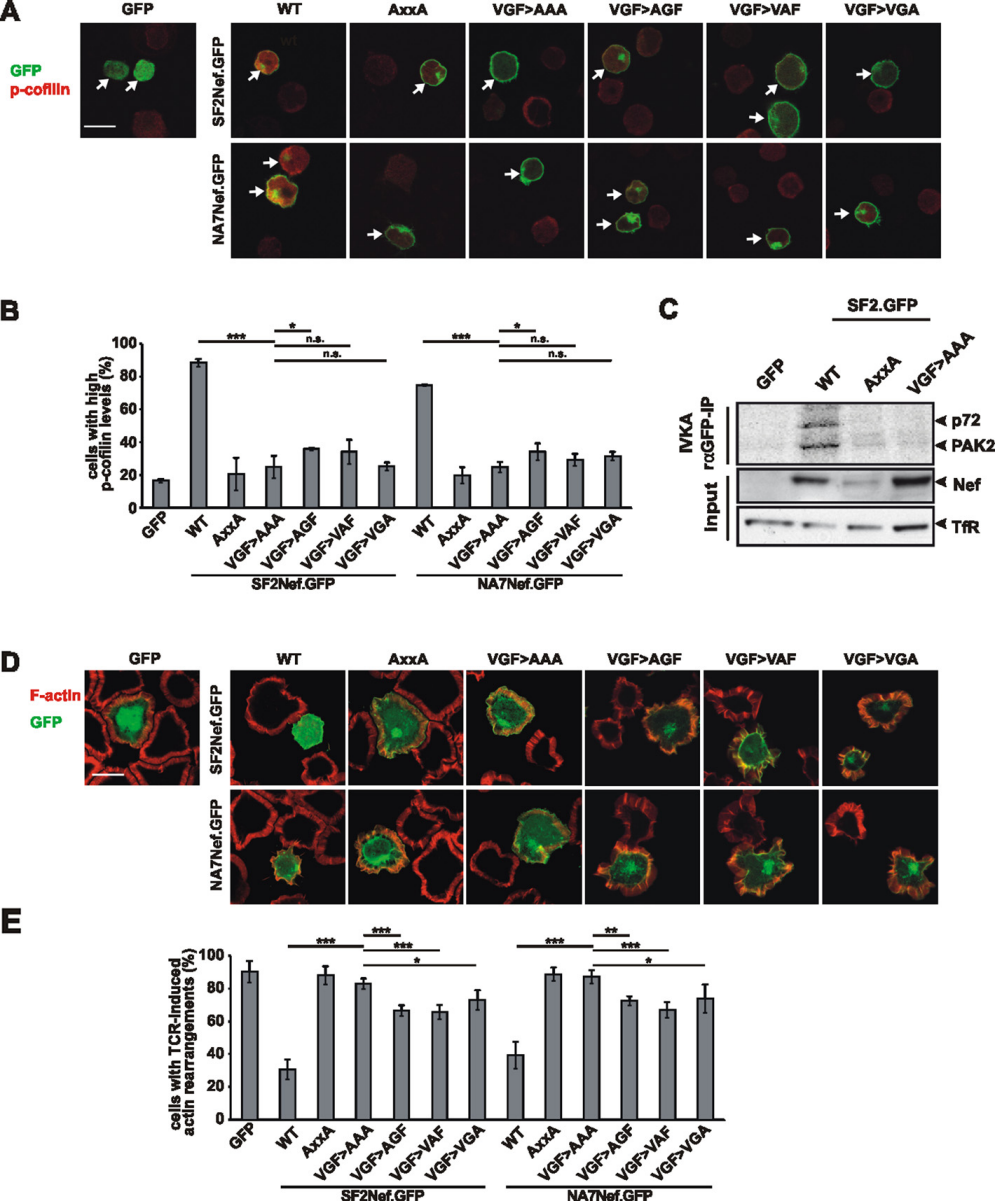
** The VGF region is important for Nef-PAK2 association, cofilin hyper- phopsorylation and inhibition of F-actin rich circumferential ring formation by Nef.** (A) Jurkat TAg cells were transfected arrows with WT, AxxA, VGF→AAA, VGF→AGF, VGF→VAF or VGF→VGA mutant Nef.GFP fusion proteins, plated onto poly-L-lysine (PLL)–coated cover glasses, fixed and stained for phospho-cofilin (p-cofilin). Images show intense staining for p-cofilin (red-orange) only in cells expressing Nef (green) of wild-type, but not of mutated sequence. Scale bar = 10 μm (B) Frequency of the cells from cultures as shown in panel A that were scored high in p-cofilin. Values are the means of 3 independent experiments, and error bars represent SD from the mean; ≥ 100 cells were analyzed per transfection; * indicates P < 0.01, ** indicates P<0.001 and *** indicates P < 0.0001. (C) Lysates of Jurkat TAg cells expressing eGFP or WT, VGF→AAA or AxxA SF2 Nef.GFP fusion proteins. Lower panels (input) show Western blot for Nef and loading control (transferrin receptor). These lysates were immunoprecipitated for Nef.GFP (rαGFP-IP), and subsequently used for *in vitro* kinase assay (IVKA). Nef-PAK2 association is detected in the IVKA only with WT Nef, by the presence of autophosphorylated PAK2 (62 kDa; p-PAK2) as well as of a yet unindentified 72 kDa substrate (p72). (D) Jurkat TAg cells were transfected with WT, AxxA, VGF→AAA, VGF→AGF, VGF→VAF or VGF→VGA mutant Nef.GFP fusion proteins, plated onto anti-CD3–coated cover glasses, fixed and stained with phalloidin to reveal F-actin (red). Scale bar = 10 μm (E) Frequency of the cells from cultures as shown in panel A that to form F-actin–rich circumferential rings. Values are the means of 3 independent experiments, and error bars represent SD from the mean; ≥ 100 cells were analyzed per transfection, * indicate P < 0.01, ** indicates P<0.001 and *** indicates P < 0.0001.

Due to the spatial proximity of the VGF motif to the polyproline stretch containing the PxxP-motif, we wondered whether both motifs support distinct functions of Nef or are integrated into one larger protein binding surface. To address this point we analyzed Nef functions in addition to MHC-I and CXCR4 downregulation known to depend on the PxxP motif and focused first on the association of Nef with the cellular p21-associated kinase 2 (PAK2) [24]. Nef-PAK2 association occurs in a highly labile multiprotein complex of about 1 MDa and causes the phosphorylation and thereby inactivation of the actin severing factor cofilin [25,26]. While the instability of the Nef-PAK2 complex limits the reliability of biochemical quantification of this association between different *nef* alleles [24,26,27], the functional consequences including PAK2-dependent deregulation of cofilin and inhibition of actin dynamics can readily be quantified and are conserved among most Nef isolates [26,28]. We therefore analyzed the ability of Nef VGF→AAA mutants from SF2 and NA7 Nef to induce hyper-phosphorylation of cofilin downstream of PAK2, using a previously established single cell-based immunofluorescence quantification assay [20,21]. As expected, cofilin was hyper-phosphorylated in Jurkat T cells expressing WT SF2 and NA-7 Nef, but not in the presence of their AxxA mutants. Similarly, VGF→AAA mutated Nef failed to induce cofilin phosphorylation (Figure 5A and 5B), suggesting that the VGF motif is essential for Nef-PAK2 association. To address this point directly, we performed an *in vitro* kinase assay to analyze the association of Nef with PAK2 activity. GFP-tagged SF2 Nef was used for this analysis due to more robust kinase signal detected with this Nef allele. Nef.GFP was immunoprecipitated from transiently transfected Jurkat T cells and subsequently subjected to an *in vitro* kinase assay, in which Nef-PAK2 association is detected by the presence of autophosphorylated PAK2 (62 kDa; p-PAK2) as well as of a yet unindentified 72 kDa substrate (p72). Expectedly [26,29], WT SF2 Nef but not its AxxA mutant efficiently associated with autophosphorylating PAK2; however, SF2 AxxA Nef was also expressed to lower levels than the WT in the experiment shown. In line with the results obtained from the cofilin-phosphorylation analyses, the VGF Nef mutant failed to associate with PAK2 activity despite robust expression and efficient immune isolation (Figure 5C). Since the deregulation of cofilin by Nef results in impaired host cell actin remodeling following external stimulation, we next analyzed the role of the VGF motif for the Nef-dependent inhibition of actin remodeling following TCR-signaling [28,30,31]. T cells transiently expressing GFP-tagged versions of WT, AxxA or VGF→AAA mutants from SF2 or NA7 Nef were plated onto anti-CD3 coated cover glasses and analyzed for the formation of circumferential F-actin rich rings by immuno fluorescence analysis. Expectedly, WT Nef expressing cells interfered with actin remodeling and prevented the formation of F-actin rich circumferential rings: as shown in Figure 4D, no actin ring is visible around green cells expressing WT Nef. In contrast, AxxA and VGF→AAA Nef variants were not able to interfere with actin polymerization (Figure 5D and 5E), so a clear circumferential staining is visible, despite Nef mutant protein expression.

Similar to the VGF→AAA mutants, single mutants of the VGF motif (AGF, VAF and VGA) were significantly reduced in their effect on cofilin phosphorylation (Figure 5A and 5B) and actin polymerization (Figure 5D and 5E). It should, however, be noted that these single mutants had a slight but significant residual effect as compared to the triple mutant, indicating that some residual activity remains for the alleles, especially for the VGF→AGF and VGF→VAF mutants. Nevertheless, these results demonstrate that the VGF motif in different *nef* alleles is critical for Nef-PAK2 association and thus consequential for Nef-mediated cofilin deregulation and disruption of host cell actin remodelling.

**Figure 6:**
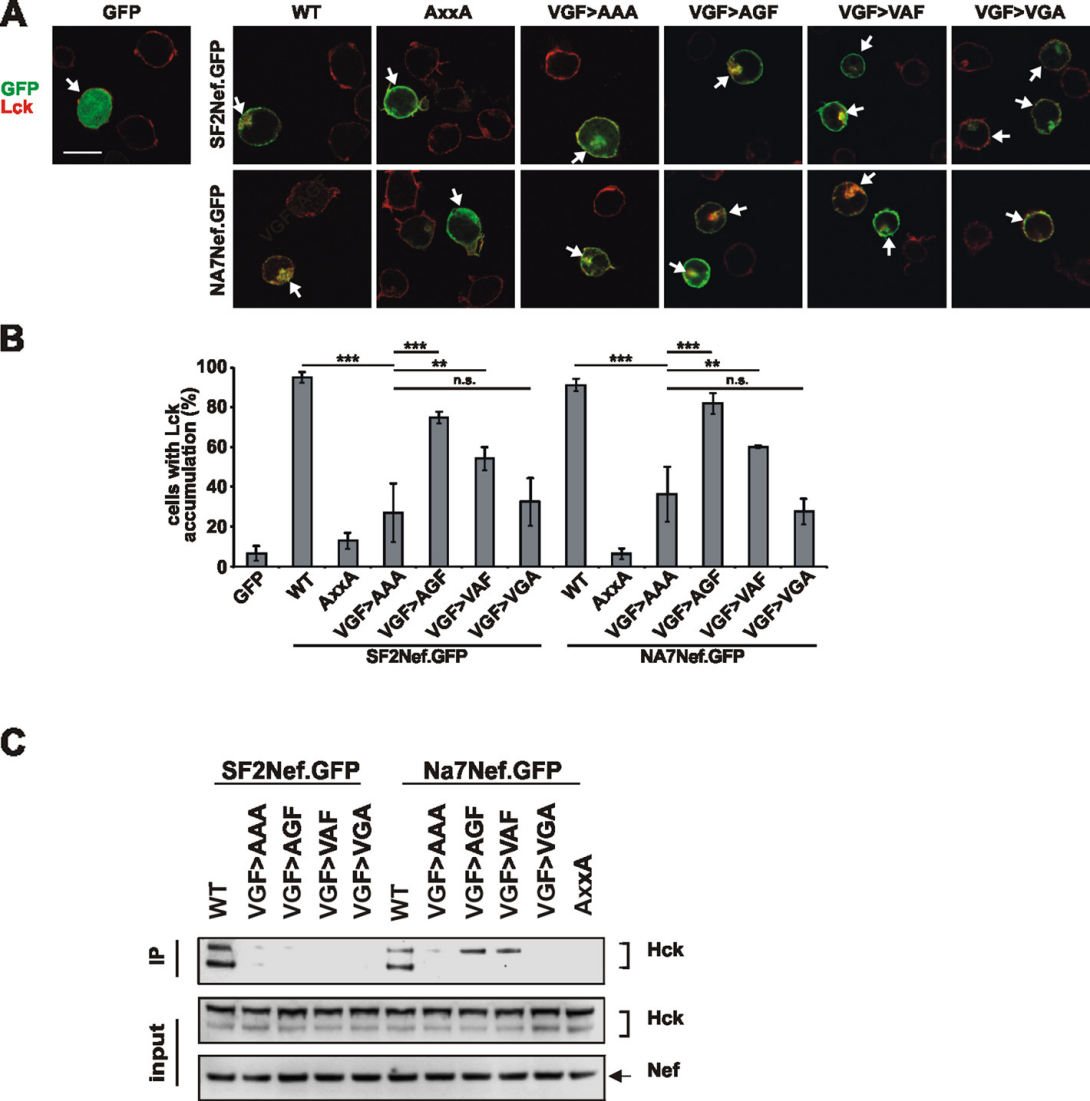
** The VGF region is important for the interaction of Nef with Lck and Hck.** (A) Jurkat TAg cells were transfected arrows with WT, AxxA, VGF→AAA, VGF→AGF, VGF→VAF or VGF→VGA mutant Nef.GFP fusion proteins, plated onto poly-L-lysine (PLL)–coated cover glasses, fixed and stained for Lck (red): wild-type but not AxxA Nef induces Lck accumulation (arrow heads), Images shown are representative for all cells analyzed. Scale bar = 10 μm (B) Frequency of the cells from cultures as shown in panel A that show Lck accumulation. Values are the means of 3 independent experiments, and error bars represent SD from the mean; ≥ 100 cells were analyzed per transfection, ** indicates P < 0.001 and *** indicates P < 0.0001. (C) Western blot of lysates of 293 T cells co-expressing WT, VGF→AAA, VGF→AGF, VGF→VAF, VGF→VGA or AxxA SF2 or NA-7 Nef.GFP fusion proteins (all similar fraction of cells positive for GFP) or GFP only together with Hck. Lower panels (input) show staining for Nef (arrow) and Hck; upper panel shows anti-GFP immunoprecipitation (IP) stained for Hck. Interaction of Nef with Hck was detected as the presence of Hck isoforms in the immunoprecipitate.

The above results demonstrated for different *nef* alleles a requirement for both the PxxP and the VGF motif in Nef-PAK2 association and cell surface receptor down modulation. All of these activities, however, also depend on additional protein interaction motifs in Nef such as an intact protein interaction surface around aa191/195 or the acidic cluster [32] and thus did not allow us to conclude unambiguously whether the VGF and PxxP motifs exert independent functions or synergize in a common molecular interaction. We, therefore, assessed next the role of the VGF motif in Nef-mediated targeting of the TCR proximal kinase Lck to the trans-golgi network (TGN), a function of Nef for which so far no protein interaction surface in Nef other than the the PxxP motif has been implicated [30,33,34]. Jurkat T cells transiently expressing GFP-tagged Nef proteins were analyzed for TGN accumulation of Lck by immunofluorescence analysis. While Lck appeared mostly at the plasma membrane with a minor intracellular fraction in GFP- and Nef AxxA-expressing cells, WT Nef expression resulted in a pronounced intracellular accumulation indicative of TGN targeting of the kinase (Figure 6A) as described previously [33]. VGF→AAA Nef mutants displayed intermediate activity in this assay and failed to affect the subcellular localization of Lck in most cells; however, some cells still showed slight TGN recruitment of Lck (Figures 6A and 6B). In contrast, single substitutions of the valine (VGF→AGF) and the glycine (VGF→VAF) respectively did not or did marginally affect Lck accumulation. However, mutation of the phenylalanine, (VGF→VGA) was sufficient to abrogate this Nef function similar to the VGF→AAA mutant, indicating that Lck accumulation by Nef depends at least in part on the VGF motif and that the phenylalanine which directly precedes the proline-rich stretch is crucial for this.

To address whether the VGF motif is directly involved in protein interactions of the adjacent PxxP motif, we probed its impact on the direct interaction of Nef with its best characterized and highest affinity PxxP ligand, the Src-family kinase Hck [35]. Nef and Hck were co-expressed in 293 T cells and Nef.GFP was immunoprecipitated. Subsequently, Co-precipitating Hck was detected by Western Blot. While different isoforms of Hck co-precipitated efficiently with WT (NA-7 or SF2) Nef, the AxxA and VGF→AAA Nef mutants were completely defective in Hck binding. Single amino acid substitution mutants did not bind the low molecular weight isoform as WT preferentially did, although some residual binding was sometimes observed with the high molecular weight isoform (Figure 6C). Together, we concluded that the integrity of the VGF motif is essential for most, if not all, functions attributed to the PxxP motif and that both motifs may be part of a common interaction surface.

### The VGF motif is essential for the effect of Nef on viral infectivity and replication

Nef has been shown to positively affect the replication of HIV *in vivo*[2] as well as in cultures of primary cells [36-38] and *ex vivo* human lymphoid tissue [39]. Multiple studies have shown that Nef positively affects viral replication by enhancing viral release and infectivity [10]. Since these functions critically rely on the PxxP motif, we addressed the replication and infectivity of VGF→AAA mutant HIV. We constructed a panel of replication competent HIV_NL4.3_-based reporter viruses expressing NL4.3 WT, AxxA and VGF→AAA Nef. These viruses express Nef together with mouse-derived heat stable antigen (HSA) from a single bicistronic mRNA (HSA-IRES-Nef) [40]. It was shown before that key activities of Nef such as enhancement of virion infectivity and increase of viral replication remain conserved in such chimeric viruses and that marker gene expression correlates with Nef expression in productively infected cells [41,42]. As control, we also constructed a Nef_stop_ HIV reporter virus, which does not express Nef due to the insertion of multiple premature stop codons [43].

**Figure 7:**
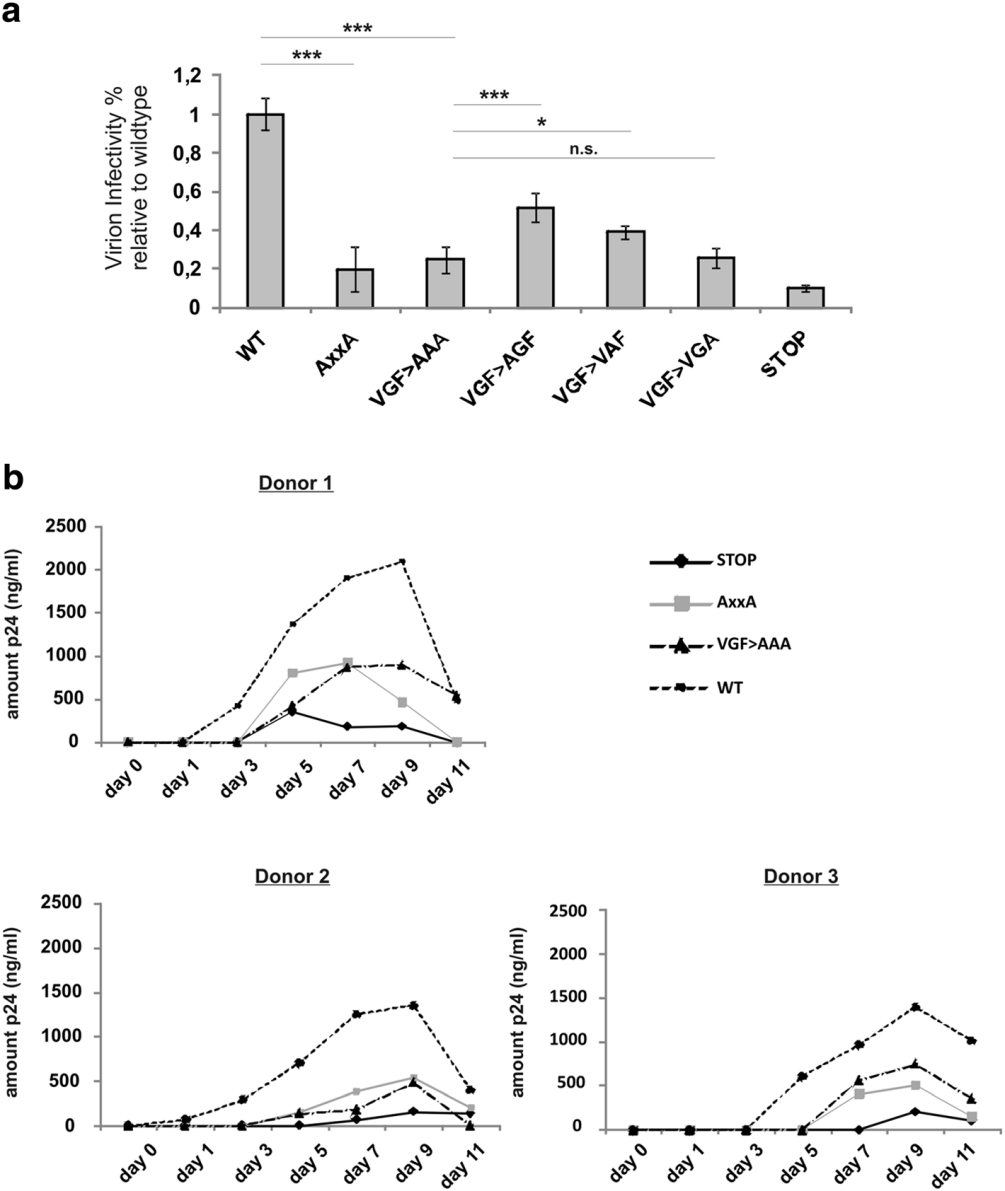
** Integrity of the VGF region is required for enhancement of virus infectivity and replication by Nef**. Replication competent HIV-1 NL4.3 HSA-IRES-Nef reporter viruses (either Nef WT, AxxA, VGF→AAA, VGF→AGF, VGF→VAF, VGF→VGA, or Stop) were produced in 293 T cells and quantified by p24 measurement (A) Enhancement of virion infectivity by different Nef mutants in HeLa P4R5 LTR-β-galactosidase indicator cells. Percentage β-gal levels were calculated relative to wild-type HIV-1 NL4-3 HSA-IRES Nef. Values are the means of 3 independent experiments, and error bars represent SD from the mean; * indicates P < 0.05, ** indicates P < 0.01 and *** indicates P < 0.001. (B) Replication kinetics in human primary CD4+ T cells. Freshly isolated peripheral blood CD4+ T cells were stimulated for 48 h with IL-2/PHA before infection. After 1, 3, 5, 7, 9 and 11 days of culture, HIV replication was monitored by determining the amount of p24 antigen in the supernatants. Plots show the replication kinetics of the HIV reporter virus in CD4+ T cells derived from 3 different donors.

We evaluated whether the VGF→AAA mutation affected the infectivity of HIV particles in HeLa P4R5 β-galactosidase indicator cells. Cells were infected with virus preparations corresponding to equal amounts of p24 and HIV-infection was quantified on the basis of β-galactosidase activity, 48 h post infection (Figure 7A). As expected, the Nef_stop_ HIV, deficient in Nef expression was almost 8-fold less infectious than HIV-1 WT virions. Both the VGF→AAA and AxxA Nef mutated HIV were severely less infective. Results shown were obtained with the NL4-3 *nef* allele and similar reduction in infectivity was seen using SF2 and NA-7 *nef* alleles (data not shown). Similary to the VGF→AAA mutant of NL4.3 Nef, single mutants were significantly less infective relative to the wt virus. Of note, single substitution mutants of the valine (VGF→AGF) and the glycine (VGF→VAF), but not of the phenylalanine (VGF→VGA), were slightly more infective than the triple mutation, resulting in an intermediate phenotype.

As a final functional correlate, we evaluated if the integrity of the VGF motif is essential for Nef enhanced HIV replication in peripheral blood lymphocytes (PBL). CD4+ T cells were isolated and stimulated with IL-2/PHA, prior to infection with isogenic viruses. Cells were infected with 293 T-derived viral supernatants corresponding to the same amount of p24. After 1, 3, 5, 7, 9 and 11 days of culture, HIV replication was monitored by determining the amount of p24 antigen in the supernatants (Figure 7B). WT NL4.3 HIV-1 replicated much more efficiently in IL-2 stimulated PBL’s relative to the *nef* stop variant. As expected the mutants of the PxxP motif were less efficient in enhancing viral replication [44,45]. Similarly, the VGF mutated Nef HIV replicated less efficiently compared to WT HIV, although the effect was attenuated in some donors when compared to the PxxP Nef mutated HIV. All donors tested showed a significantly reduced replication rate of HIV VGF→AAA Nef mutated virus compared to WT Nef HIV. After day 9 of culture, viability in infected cultures decreased significantly, explaining the general drop in p24 levels at day 11. As a control, we also evaluated the amount of HSA expressing cells at several time points by flow cytometry as an alternative measure for HIV expression, with similar results (data not shown).

## Discussion

Although not essential for viral replication, HIV-1 Nef is important for disease progression and, therefore, is considered a pathogenic factor in primate lentiviridae [10]. For its function, Nef depends on specific surfaces to interact with host cell proteins. Therefore, a strong positive selection pressure exists to keep the property of these surfaces conserved [46]. It is key to identify conserved sites in Nef with functional importance in protein-protein interactions, since they might serve as potential target sites for pharmacological intervention.

In the present study, we identified, by functional analysis of a panel of clinical HIV-1 and HIV-2 *nef* alleles, a previously unrecognized conserved region. A HIV-1 group O *nef* allele, was mutated in an amphipathic stretch of amino acids located in the core domain encompassing the acidic cluster, a VGF region and the PxxP motif, resulting in the loss of downregulation of MHC-I and CXCR4, but not of CD4. By selective mutation of this O8 *nef* allele back to the consensus sequence, we show that both an intact PxxP motif in conjunction with an intact VGF region are needed to restore the defect in receptor trafficking. We could extend this observation by showing that the VGF region is essential for the association of Nef with active PAK2 and consequently hyper-phosphorylation of cofilin resulting in the inhibition of actin remodeling following TCR triggering. In addition, Nef-induced Lck accumulation in the TGN also requires integrity of the VGF region. The importance of the proline-rich region for this Nef function has been studied extensively [10,47]. However, despite its high conservation in HIV-1 and SIVcpz *nef* alleles, the function of the VGF region remained poorly investigated.

Modulation of cell surface molecules such as MHC-I and chemokine receptors or the TNF receptor-associated factor TRAF2 were shown to depend on the conservation of the PxxP motif as well as the acidic cluster (EEEE) [19,48,49]. How the acidic cluster contributes to downregulation of MHC-I is controversial. Several studies have observed that the four contiguous glutamate residues interact with PACS-1 and PACS-2 to initiate assembly of a multiprotein complex which targets MHC-I to the trans-Golgi network for subsequent degradation [50,51]. However, in an alternative model for MHC-I downregulation, in which Nef interacts with both MHC-I and the μ subunit of the AP-1 endosomal coat complex, the EEEE motif plays only a stabilizing role [52,53]. The PxxP motif on the other hand is essential for the interaction of Nef with Src kinases (like Hck); misrouting of Lck as well as association of Nef with the cellular kinase PAK2, results in elevated cellular levels of inactivated phosphorylated cofilin, a deregulation that is instrumental for the inhibition of TCR- or chemokine-induced F-actin remodeling [26,28-31,34].

Triple mutants of the VGF motif (VGF→AAA) lost both ‘trafficking’ (MHC-I, CXCR4) as well as ‘signaling' (PAK2 association, cofilin hyper-phosphorylation and inhibition of actin ring formation) functions, suggesting that the VGF region is functionally linked with the PxxP motif. Moreover, Baugh *et al*. showed that similar to the PxxP motif, MHC-I downregulation, interaction with PAK2, and to a lesser degree enhancement of virion infectivity are dependent on the acidic cluster [32]. Our observation that mutation of VGF into triple alanines similarly abrogates these multiple effects of Nef is a hint that the acidic cluster-VGF-PxxP triplet forms a functional unit important for protein-protein interactions with host cell factors.

In the dysfunctional Nef O8 allele, the VGF motif is in fact deleted, but not replaced to varying amino acids by gene tropism. The effect of the deletion mutation could therefore be indirect, meaning that only the correct spacing between the acidic cluster EEEE motif and the PxxP motif is required for Nef functionality, *i.e.* the correct positioning of the EEEE motif and potentially the preceding CAWL protease recognition motif with respect to the Nef core domain. Alternatively, the effect of Nef dysfunction could be direct through binding of these amino acids to the primary receptors itself or to any stimulatory co-factor required for receptor downregulation. As mutation of the VGF motif to AAA in O8 Nef did not restore the Nef trafficking functions, the effect of mutation is most likely direct. Our finding that individual mutation of the three amino acids (AGF, VAF, VGA) is sufficient to interfere with the effect of Nef on receptor trafficking and TCR signaling further supports this hypothesis and shows that the function of V-G-F is sequence specific. The integrity of the phenylalanine appears to be specifically essential for functions related to the proline-rich motif. In contrast to single mutants of the valine and the glycine (VGF→AGF and VGF→VAF), single mutants of this amino-acid (VGF→VGA) did not accumulate Lck. Of note, several SH3 binding regions of mammalian proteins contain a N-terminal phenylalanine, which is required for efficient ligand binding [54]. These observations suggest that the effect of Nef on TCR induced actin remodeling and on Lck targeting to the TGN are mechanistically different, as was shown before [30].

Shelton *et al.* showed recently that the VGFPV region, in their study identified as the secretion modification region, forms a specific binding surface for mortalin which subsequently promotes cellular secretion of extracellular Nef [55]. The phenotypes which we analysed in this study are all cell intrinsic and are unlikely to be explained by differences in levels of extracellular Nef. The results of Shelton *et al*. and ours together show that the VGF region is not just a mere spacer between the acidic cluster and the proline rich region. Instead, it acts as an interaction surface, which together with its neighboring motifs forms a functional unit.

Replication of HIV-1 in primary T lymphocytes is tightly coupled to their activation state. While HIV-1 undergoes early replication events in quiescent CD4+ T cells, subsequent steps in the viral life cycle require T cell activation. One of the prime roles of Nef *in vivo* is to fine tune activation states in infected T cells [56]. Nef interferes with TCR proximal signaling to prevent T cell activation and activation–induced cell death upon antigenic stimulation [28,30,31,55], while at the same time enhancing distal TCR signaling effects to promote proviral expression [33,57,58]. Both functions of Nef depend on the interaction of several specific SH3-domain containing host cell proteins with the conserved PxxP motif. For example, the PxxP motif interacts with the SH3-domain of Lck, misrouting the TCR proximal kinase Lck from the plasma membrane to the trans Golgi-network (TGN), altering TCR proximal signaling events [28,30,33,59]. In our HIV replication assay, WT HIV viruses replicated much more efficiently then HIV Nefstop viruses. The requirement of PxxP residues for efficient HIV-1 replication in T-cells is controversial [35,39,44,59]. Lundquist and coworkers did not observe significant differences between the replication-efficiency of WT, AxxA mutant and polyproline deleted viruses [60], while Saksela and co-workers reported that AxxA mutant replicate as delta-Nef viruses [35]. In our hands, the replication of the HIV Nef AxxA viruses was significantly attenuated compared to WT viruses. The fact that we use a different proviral backbone and other culture methods for CD4^+^ lymphocyte stimulation may account for the discrepancy between our results and observations from others. Similar to Nef AxxA viruses, VGF→AAA mutants replicated much less efficiently than WT HIV viruses. We recently showed that the accumulation of Lck is required for TGN-associated Ras-Erk signaling, which in turn was shown to promote IL-2 production and enhance virus spread [33]. As HIV viruses harboring AxxA or VGF→AAA mutations in Nef fail to efficiently accumulate Lck [30], this could explain the altered replication kinetics of these mutants. Interestingly, our previous observation that the HIV O8 isolate showed reduced fitness compared to other isolates of the same group might be due to the deletion of the VGF domain [16].

Despite the opposing findings concerning the role of the PxxP motif for replication of HIV, there is general consensus that this domain is required for Nef to enhance the infectivity of HIV virions [61-63]. The mechanism by which Nef enhances viral infectivity remains unclear, but is thought to involve alterations of specific signaling and trafficking events in the producer cell. [9]. We observed that, in line with our other results, VGF→AAA mutants were similarly less infective than wild type viruses.

In our study, we only analyzed the importance of the VGF motif in Nef for HIV-1 function. Despite VGF conservation in HIV-1 and SIV_cpz_; HIV-2 and SIV_smm_ instead harbor VGV. There are no indications that this change would result in major functional differences, since PxxP dependent functions such as downregulation of MHC-I, accumulation of Lck, deregulation of actin remodeling and association with PAK2 are well conserved between both HIV-1 Nef and HIV-2 Nef [26,28]. Moreover, both phenylalanine and valine are hydrophobic amino acids, conserving the lipophilic character of the entire VGF/V region, and they are not expected to impose a different ultrastructure that would imply different host protein interactions. Rather, the strong conservation of VGF/VGV in HIV/SIV Nef suggests that such a structural motif is required for function of acidic cluster-VGF/V-PxxP motifs in general.

## Conclusions

Our results extend the previous research performed on the function of the PxxP motif. Based on our and earlier observations from other groups, we propose a new model in which the PxxP motif operates in the context of a larger amphipathic protein surface, encompassing both the acidic cluster and the VGF linker region. Moreover, integrity of the VGF region is required for SH3 binding and likely for subsequent formation of multi-protein complexes. The fact that a multitude of Nef functions required for enhancement of viral replication and disease progression are dependent on the formation of these protein complexes suggests that the amphipathic region could serve as a prime target for pharmaceutical intervention.

### Availability of supporting data

Nucleotide sequences of isolated HIV-1 and HIV-2 Nef alleles used in this study were submitted to Public sequence repository (Genbank accession numbers JQ 990942 - JQ990960).

## Methods

### Viruses and Nef alleles

HIV-1 group M and group O isolates were obtained from the AIDS Research and Reference Reagent Program, while the HIV-2 strains were previously isolated from patients attending the AIDS clinic at the Institute of Tropical Medicine in Antwerp, Belgium, with the approval of the ethical committee. Virus stocks were propagated and expanded in short-term cultures of PBMCs treated with PHA (1 μg/mL) and IL-2 (10 ng/mL). Genomic DNA was extracted using the Qiagen Blood kit (Qiagen, Venlo, The Netherlands) according to the manufacturer’s instructions. Nef alleles from different HIV subtypes were amplified by semi-nested polymerase chain reaction (PCR) with Platinum Pfx DNA polymerase (Invitrogen, Merelbeke, Belgium). Primers recognizing Env and the 3’LTR regions of the HIV genome used for the first round of PCR were forward: 5’-GCACTCAAGGCAAGCTTTATTGAGGC-3’ and reverse 5’-CCACATACCTAGAAGAATAAGACAGG-3’. A nested PCR reaction was performed after sequencing and using allele-specific forward and reverse primers covering the complete nef coding sequence and containing restriction sites for BamHI and EcoRI, respectively (see Additional file 1: Table S1A). Site specific mutants of Nef O8, VI1422, B2 and NA-7 in LZRS-IRES-eGFP were generated with PCR using allele specific primers described in Additional file 1: Table S1B and Table S1C.

### Plasmid construction

Nef amplicons were cloned into the LZRS-IRES-eGFP retroviral vector, using BamHI and EcoRI restrictions sites [64]. Plasmids were purified with the Qiaprep Miniprep kit (Qiagen). The integrity of the constructs and the *nef* genes was confirmed by direct sequencing (ABI, Foster City, California). Alignments of the *nef* sequences together with selected reference sequences were performed using BioEdit version 5.0.9. Type, group and subtype designation was confirmed by constructing phylogenetic trees by the neighbor joining method using MEGA 4 [65] (see Additional file 1: Figure S1).

Expression constructs for GFP fusion proteins of HIV-1 SF2 Nef, HIV-1 NA-7 Nef and SF2 AxxA were already described and were constructed by cloning PCR fragments of the respective *nef* genes into pEGFP-N1 (Clontech) under the control of the cytomegalovirus promoter [26,31]. The expression constructs for NA7 Nef VGF → AAA and SF2 Nef VGF → AAA and NA-7 AxxA were generated by site directed mutagenesis from the wild-type (WT) plasmid (primers Additional file 1: Table S1D).

The proviral constructs used for HIV infectivity and replication assays are derived from the NLENG1-IRES vector [66], which expresses HSA-IRES-Nef cloned in HIV-1 NL4-3 provirus, as described before [40]. These constructs express from the nef reading frame (together with Nef as two individual proteins) mouse heat-stable antigen as a protein reporter. A BamH1/NgoMIV fragment corresponding to the second exon of *tat* to the end of *nef* was cut out from the vector and was ligated into pSUPER [67]. Next, SF2 and NA-7 *nef* alleles and their specific AxxA and VGF mutants were amplified by PCR using primers containing restriction sites for BstXI and BspEI from the pEGFP-N1 vectors described above. A *nef* allele, with deleted initiation codon, two in frame stop codons at amino acid positions 4 and 5 and premature stop codons at positions 73 and 74 in the *nef* ORF, disrupting the *nef* gene was generated by standard PCR and cloning techniques, similarly to what was described before (Nef_stop_) [43]. The amplified *nef* alleles were subsequently cut with BstXI and BspE and ligated into the pSUPER plasmid, swapping it with the WT *nef* present. NL4.3 AxxA and VGF→AAA mutants were generated based on the pSUPER vectors by site directed mutagenesis. Next, BamH1/NgoMIV fragments of newly constructed pSUPER plasmids were ligated into the NL4-3 HSA-IRES-Nef proviral construct. All these final proviral constructs were verified for correct *nef* allele sequence by direct sequencing.

The vector for the 764 amino acid p59Hck-mCherry fusion protein was constructed by PCR amplifying human Hck cDNA insert from the image clone 4855747 using primers containing BglII (sense) and KpnI (antisense), and inserting it between the BamHI and KpnI sites of the pEBB vector [68], followed by cloning of red fluorescent protein mCherry (Clontech) between the KpnI and EagI sites after the p59Hck insert.

### Cell lines, transfection, transduction and flow cytometry

Jurkat TAg [26,29] and Jurkat CD4-CCR5 (Programme EVA Centre for AIDS Reagents, NIBSC, U.K.), are both Jurkat E6.1-derived cell lines. The easy to transfect Jurkat TAg cells express the large T antigen of simian virus 40 and were grown in RPMI 1640 medium supplemented with 10% fetal calf serum (FCS, Hyclone), 100 U/mL penicillin, and 100 g/mL streptomycin (both from Gibco). Jurkat CD4-CCR5 cells were grown in IMDM medium supplemented with 2 mM L-glutamin, 10% heat-inactivated fetal calf serum (FCS), 293 T cells (American Type Culture Collection (ATCC) Manassas, VA) were maintained in IMDM medium, supplemented with 10% FCS, 2 mM L-glutamin, 100 U/mL penicillin, and 100 U/mL streptomycin.

Mouse antihuman monoclonal antibodies used were: CD4 (clone SK3; [PE] and [APC]), anti-CXCR4, anti-MHC-I [PE] (anti-human histocompatibility leukocyte antigens [HLA]-A,-B,-C), all purchased from Becton Dickinson Immunocytometry systems (Beckton Dickinson, Erembodegem, Belgium) and anti-mouse-CD24-APC (HSA, heat stable antigen; BioLegend, San Diego, CA). The cells were analyzed on a FACSCalibur flow cytometer (Becton Dickinson). Forward light scattering, orthogonal scattering, and fluorescence signals were stored and analyzed using the CellQuest software (Becton Dickinson).

Cells were transfected using either calcium-phosphate precipitation (Invitrogen, Merelbeke, Belgium) or JetPei (Polyplus, Sélestat, France) according to the description of the manufacturer. For transduction of suspension cell lines, cells were mixed with retroviral supernatant, pre-incubated with DOTAP (Roche diagnostics). To increase transduction efficiency, cells were centrifuged (90 minutes, 950 g, 32°C) [64].

### Production of retroviral vectors and replication competent HIV

To produce retroviral vectors, the Phoenix-Amphotropic packaging cell line was transfected with LZRS-IRES-eGFP (control) and LZRS-Nef-IRES-eGFP plasmids using calcium-phosphate precipitation as previously described [17,64,69].

Replication competent HIV was produced by transfecting 293 T cells with the HIV-1 NL4-3 –HSA-IRES-Nef proviral constructs described above using the JetPEI method. The medium was changed after overnight incubation, and virus was harvested 24 h later and stored at −80°C. The content of viral p24 antigen was quantified using a HIV p24 enzyme-linked immunosorbent assay kit (Innogenetics, Ghent, Belgium).

### Immunofluorescence

Immunofluorescence stainings were performed as previously described [26,28]. Briefly, for staining of F-actin (0.5 ng/ml phalloidin-TRITC), cells were fixed for 15 min with 3,7% paraformaldehyde (PFA)/phosphate-buffered saline (PBS), permeabilized with 0.1% Triton X-100/PBS for 2 min and subsequently blocked for unspecific binding with 1% bovine serum albumin (BSA)/PBS for 15 min. Hoechst 33258 was used in a concentration of 1 ng/ml. For staining of phospho-cofilin (p-cofilin) (cell signaling) (1:50), and Lck (Santa Cruz) (1:50) all solutions, dilutions, and wash steps were done with Tris-buffered saline (TBS; 50 mM Tris, 150 mM NaCl [pH 7.5]). Blocking was performed for 30 min, and incubation was performed with the first antibody overnight at 4°C. Confocal pictures of p-cofilin in Jurkat T cells were acquired using a Zeiss LSM 510 Axiovert microscope and LSM Meta software. Images were processed using Adobe Photoshop CS3.

### In vitro kinase assay

*In vitro* kinase assays (IVKA) were essentially performed as described previously [26]. Briefly, Jurkat T lymphocytes were transfected with expression plasmids for Nef.eGFP. After 24 h, cells were lysed in KEB (137 mM NaCl, 50 mM Tris/HCl [pH 8], 2 mM EDTA, 0.5% Nonindet P-40, and protease inhibitors) supplemented with Na3VO4, and cleared lysates were sampled for Western blot and immunoprecipitated with a rabbit anti-GFP antibody. After intensive washing in KEB, the immunoprecipitates were resuspended in KAB (50 mM HEPES [pH 8], 150 mM NaCl, 5 mM EDTA, 0.02% Triton X-100, 10 mM MgCl2) containing 10 μCi of [γ-^32^P]ATP per reaction. After incubation for 10 min, samples were washed, and bound proteins were separated by SDS-PAGE and subjected to autoradiography. The kinase signal was quantified relative to the SF2 *nef* allele using Quantity One (Bio-Rad).

### Co-immunoprecipitation

For the Nef–Hck co-immunoprecipitation experiments, 293 T cells were transfected with expression plasmids for Nef.eGFP and p59Hck (Cherry fusion protein). After 24 h, cells were lysed in KEB (137 mM NaCl, 50 mM Tris/HCl [pH 8], 2 mM EDTA, 0.5% Nonidet P-40, and protease inhibitors) supplemented with Na3VO4, and cleared lysates were immunoprecipitated with a GFP-Trap (Chromotek, Planegg-Martinsried, Germany). After intensive washing in KEB, the precipitate was incubated in anti-Hck Santa Cruz Biotechnology inc. Santa Cruz, US), diluted 1:1000 in 50% blocking buffer.

### Western blotting

Nef transduced Jurkat CD4-CCR5 cells sorted for eGFP expression, were lysed in laemmli sample buffer. Equal amounts of protein (40 μg) were run on a 12% precast NuPAGE 4-12% Bis-Tris polyacrylamide gel (Invitrogen) in reducing conditions and blotted onto a polyvinyldifluoride (PVDF) membrane (Invitrogen). Primary antibodies used were sheep-anti-Nef antiserum (Programme EVA Centre for AIDS Reagents, NIBSC, UK.). Detection was done using enhanced chemiluminescence (GE Healthcare, Chalfont St. Gilles, UK). To control that an equal amount of protein was loaded for each sample, blots were also stained for β-actin (primary anti-β-actin antibody: clone C4, ICN, Aurora, OH, USA).

### Infectivity assays

Virus infectivity was determined using P4R5 HeLa reporter cells (NIH AIDS Reference and Reagent Program) [70]. Briefly, the cells were sown out in 96-well dishes in a volume of 200 μl IMDM supplemented with 2 mM L-glutamin and 10% FCS and infected after overnight incubation with virus stocks containing 40 ng of p24 antigen (Innogenetics, Zwijnaarde, Belgium) produced by transiently transfected 293 T cells and centrifuged (90 minutes, 950 g, 32°C). 48 hours postinfection viral infectivity was detected using a beta-galactosidase screen kit from Thermo scientific (Pierce Biotechnology, Rockford, USA) as recommended by the manufacturer. β-Galactosidase activities were quantified as relative light units per second using an Orion Microplate Luminometer. To calculate percent values, relative light units per second obtained for wild-type HIV-1 NL4-3 HSA-IRES Nef infection were set to 100%.

### Viral replication in CD4+ PBL cultures

To determine the efficiency of HIV-1 replication in peripheral blood CD4^+^ lymphocytes, we isolated CD4^+^ cells from buffy coat peripheral blood mononuclear cells (normal blood donors, Red Cross, Ghent, Belgium) by negative selection using paramagnetic beads (MACS; Miltenyi Biotec, Bergish Gladbach, Germany). After isolation, the cells were cultured in RPMI medium supplemented with 2 mM L-glutamin, 10% heat-inactivated fetal calf serum, phytohemagglutinin (1 μg/mL; Thermo Fisher Scientific, Waltham, USA), 20 ng/mL IL-2 (Peprotech, Rocky Hill, USA), 100 U/mL penicillin, and 100 g/mL streptomycin. Thereafter, 1 ng of p24 antigen was added to 2,5*10^5^ PBLs, and the culture was spinoculated at 2,300 rpm for 90 minutes at 32°C. After centrifugation, the supernatant was removed and the cells were further cultured in RPMI supplemented with 20 ng/mL IL-2. HIV replication was monitored at 1, 3, 5, 7, 9 and 11 days post-infection by measuring the amount of p24 antigen present in the supernatants, using a HIV p24 enzyme-linked immunosorbent assay kit (Innogenetics, Ghent, Belgium). Alternatively, we also determined the expression of the HSA reporter gene at each time point using flow cytometry, as was described before [40].

### Statistical analysis

Data were analysed with non-parametric Mann–Whitney U test, one-tailed (SPSS, version 17; SPSS, Chicago, USA).

## **Abbreviations**

HIV, Human immunodeficiency virus; SIV, Simian immunodeficiency virus; AIDS, Acquired immunodeficiency syndrome; TCR, T cell receptor; MHC-I/II, Major histocompatibility complex class-I/ II; PAK2, P21-activated kinase 2; Lck, Lymphocyte-specific protein tyrosine kinase; TGN, Trans-golgi-network; PBL, Peripheral blood lymphocytes; IL-2, Interleukin-2; PHA, Phytohaemaglutinin A; eGFP, Enhanced green fluorescent protein; PACS-1/2, Phosphofurin acidic cluster- sorting protein-1/2; HSA, Heat stable antigen; AP-1, Adaptor protein 1; DNA, Deoxyribonucleic acid; IRES, Internal ribosomal entry site; FCS, Fetal calf serum; PE, Phycoerythrine; APC, Allophycocyanine; PFA, Paraformaldehyde; IVKA, In vitro kinase assay; PVDF, Polyvinyldifluoride.

## Competing interests

The authors declare that they have no competing interests.

## Author’s contributions

PM was responsible for the concept and design of the study, acquisition of most of the data and the writing of the the manuscript; BS was responsible for *in vitro* kinase and actin ring assays and helped with p-cofilin and Lck assays and critically revised the manuscript; EN gave substantial practical support during acquisition of the data; JV and VI optimized the HIV replication assay, helped with proviral construction and critically revised the manuscript; MG performed *in silico* modeling, gave substantial scientific input and critically revised the manuscript; KS constructed the p59-HcK expression plasmids and critically revised the manuscript; GV provided the clinical HIV1- and HIV-2 isolates and critically revised the manuscript; KKA performed *in vitro* culture of primary isolates, is responsible for design of the study and critically revised the article. OF gave substantial scientific input and critically revised the manuscript; BV was responsible for the concept and design of the study, gave substantial scientific input and wrote the manuscript. All authors read and approved the final manuscript.

## Supplementary Material

Additional file 1 **Figure S1.** Alignment of the protein sequences and evolutionary relationship of the isolated HIV-1 and HIV-2 Nef proteins. **(A)** The evolutionary history was inferred using the neighbor-joining (NJ) method as conducted in MEGA 4. **(B–E)** Amino acid sequence alignments relative to references sequences, obtained from the Los Alamos database HIV sequence database. Dots connote identity with the reference sequence, whereas individual variations are shown by the single letter amino acid code. Dashes indicate gaps introduced into the sequence to optimize the alignment. **(B)** Alignment of HIV-1 group M subtype B Nef amino acid sequences. **(C)** Alignment of HIV-1 group M subtype C Nef amino acid sequences. **(D)** Alignment of HIV-1 group O Nef amino acid sequences. **(E)** Alignment of HIV-2 Nef amino acid sequences. **Table S1. A)** Allele-specific primers covering the complete Nef coding sequence. **B)** Primers to generate site-specific mutants in Nef O8. **C)** Primers to generate VGF→AAA mutants (indicated by ΔVGF) in B2, VI1422, NA-7 and NL4.3 Nef. **D)** Primers for site specific mutagenesis of NA-7.GFP and SF2.GFP fusion proteins and NL4.3. Click here for file
