# Supplementary material for: Identification of a highly conserved valine-glycine-phenylalanine amino acid triplet required for HIV-1 Nef function
Source: Retrovirology. 2012 Apr 27;9:34. doi: 10.1186/1742-4690-9-34 (PMC3476393; doi:10.1186/1742-4690-9-34)
Supplement: Additional file 1 — Figure S1. Alignment of the protein sequences and evolutionary relationship of the isolated HIV-1 and HIV-2 Nef proteins. (A) The evolutionary history was inferred using the neighbor-joining (NJ) method as conducted in MEGA 4. (B–E) Amino acid sequence alignments relative to references sequences, obtained from the Los Alamos database HIV sequence database. Dots connote identity with the reference sequence, whereas individual variations are shown by the single letter amino acid code. Dashes indicate gaps introduced into the sequence to optimize the alignment. (B) Alignment of HIV-1 group M subtype B Nef amino acid sequences. (C) Alignment of HIV-1 group M subtype C Nef amino acid sequences. (D) Alignment of HIV-1 group O Nef amino acid sequences. (E) Alignment of HIV-2 Nef amino acid sequences. Table S1. A) Allele-specific primers covering the complete Nef coding sequence. B) Primers to generate site-specific mutants in Nef O8. C) Primers to generate VGF→AAA mutants (indicated by ΔVGF) in B2, VI1422, NA-7 and NL4.3 Nef. D) Primers for site specific mutagenesis of NA-7.GFP and SF2.GFP fusion proteins and NL4.3. [file 1742-4690-9-34-S1.doc]

**Additional Figure 1**

**Alignment of the protein sequences and evolutionary relationship of the isolated HIV-1 and HIV-2 Nef proteins.**

(A) The evolutionary history was inferred using the neighbor-joining (NJ) method as conducted in MEGA 4. (B-E) Amino acid sequence alignments relative to references sequences, obtained from the Los Alamos database HIV sequence database. Dots connote identity with the reference sequence, whereas individual variations are shown by the single letter amino acid code. Dashes indicate gaps introduced into the sequence to optimize the alignment. (B) Alignment of HIV-1 group M subtype B Nef amino acid sequences. (C) Alignment of HIV-1 group M subtype C Nef amino acid sequences. (D) Alignment of HIV-1 group O Nef amino acid sequences. (E) Alignment of HIV-2 Nef amino acid sequences.

**A**


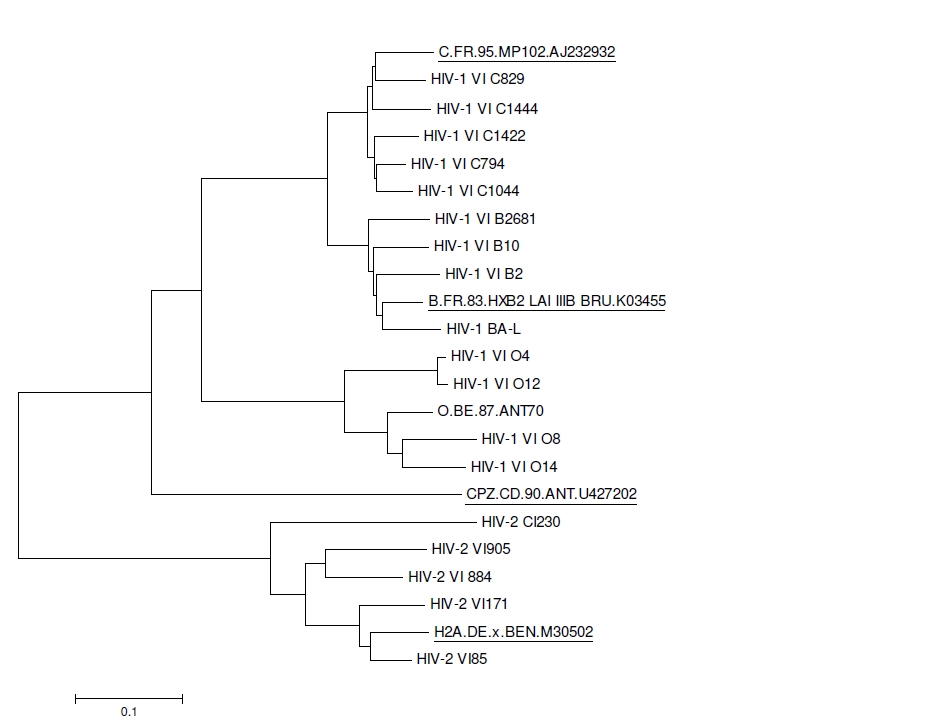


**B**

**C**

**D**

**E**

**Additional Table S1**

**A)** Allele-specific primers covering the complete Nef coding sequence

| ***Subtype*** |  | ***Nef allele*** | | ***FW primer*** | ***REV primer*** |
| --- | --- | --- | --- | --- | --- |
|  |  |  |  | |  |
| ***HIV-1 group M*** | ***(B)*** | ***B2*** | 5’-TTTGGATCCTTTTTATAAATGGGTGGCAAGTTGTCAAAAAG-3’ | | 5’-TTTGAATTCTTTACGCGTTCAGCAGTCTTTGAAGTACTCCGG-3’ |
|  |  | ***B10*** | 5’-TTTGGATCCTTTTTATAAATGGGTGGCAAGTGGTCAAAACG-3’ | | 5’-TTTGAATTCTTTACGCGTTCAGCAGTCTTTGAAGTACTCCGG-3’ |
|  |  | ***VI2681*** | 5’-TTTGGATCCTTTTTATAAATGGGAGGCAAGTGGTCAAAACG-3’ | | 5’-TTTGAATTCTTTACGCGTTCAGTCTTTGTAGAACTCCGGGTGC-3’ |
|  |  | ***BA-L*** | 5’-TTTGGATCCTTTTTATAAATGGGTGGCAAGTGGTCAAAAAG-3’ | | 5’-TTTGAATTCTTTACGCGTTCAGCAGTCTTTGTAGTATTCCGG-3’ |
|  |  |  |  | |  |
|  | ***(C)*** | ***VI1444*** | 5’-TTTTGGATCCATGGGGGCCAAATGGTCAAAATGTAG-3’ | | 5’-TTTTGAATTCTCAGCAGTTCTTCTTGTAATAGTCCGGATG-3’ |
|  |  | ***VI1044*** | 5’-TTTTGGATCCATGGGGAACAAGTGGTCAAAATGTAG-3’ | | 5’-TTTTGAATTCTCAGCAGTTCTTGTAAAACTCCGGATG-3’ |
|  |  | ***VI1422*** | 5’-TTTTGGATCCATGGGGGGCAAATGGTCAAAATGTAG-3’ | | 5’-TTTTGAATTCTCAGCAGTTTTTGTAGTACTCCGGATG-3’ |
|  |  | ***VI794*** | 5’-TTTTGGATCCATGGGGGGCAAATGGTCAAAATG-3’ | | 5’-TTTTGAATTCTCAGCAGTCTTTGTAATACTCCGGATG-3’ |
|  |  | ***VI829*** | 5’-TTTTGGATCCATGGGGGGCAAGTGGTCAAAAAGTAG-3’ | | 5’-TTTTGAATTCTCAGCAGTCTTTGTAATACTCCGGATG-3’ |
|  |  |  |  | |  |
| ***HIV-1 group O*** |  | ***O4*** | 5’-TTTGGATCCTTTTTATAAATGGGGAATGCATGGAGCAAAAG-3’ | | 5’-TTTGAATTCTTTACGCGTTTATTTGGGGAAGAGCTCTGGGTG-3’ |
|  |  | ***O8*** | 5’-TTTGGATCCTTTTTATAAATGGGAAACGCATTGAGAAAAC-3’ | | 5’-TTTGAATTCTTTACGCGTTTAGTCTTTGGAGAAGAGCTCTGG-3’ |
|  |  | ***O12*** | 5’-TTTGGATCCTTTTTATAAATGGGGAATGCATGGAGCAAAAG-3’ | | 5’-TTTGAATTCTTTACGCGTTTATTTGGGGAAGAGCTCTGGGTG-3’ |
|  |  | ***O14*** | 5’-TTTGGATCCTTTTTATAAATGGGAAACGTATTAGGGAAAAGC-3’ | | 5’-TTTGAATTCTTTACGCGTTTAGTCTTTGAGGAAGAGTTCTGG-3’ |
|  |  |  |  | |  |
| ***HIV-2*** |  | ***VI905*** | 5’-TTTGGATCCTTTTTATAAATGGGTGCGAGTGGCTCCAAGAAG-3’ | | 5’-TTTGAATTCTTTACGCGTTTAACTGAATGGTATCCCTCTTGC-3’ |
|  |  | ***CI85*** | 5’-TTTGGATCCTTTTTATAAATGGGTGCGAGTGGCTCCAAGAAG-3’ | | 5’-TTTGAATTCTTTACGCGTTTATTCACTATATGGTATCCCTC-3’ |
|  |  | ***VI171*** | 5’-TTTGGATCCTTTTTATAAGATGGGTGCCAGTGGTTCCAAGAAGC-3’ | | 5’-TTTGAATTCTTTACGCGTTTAACTATATGGTATCCCTCTTGC-3’ |
|  |  | ***VI884*** | 5’-TTTGGATCCTTTTTATAAGATGGGTGCGAGTGGTTCCAAGGAGC-3’ | | 5’-TTTGAATTCTTTACGCGTTTAACTGAAAGGTATCCCTCTTGC-3’ |
|  |  | ***CI230*** | 5’- TTTGGATCCTTTTTATAAGATGGGTGCCAGTGGTTCCAAGAAGC -3’ | | 5’-TTTGAATTCTTTACGCGTTTATTCACTATATGGTATCCCTCTTGC-3’ |
|  |  |  |  | |  |
|  |  |  |  | |  |

**B)** Primers to generate site-specific mutants in Nef O8

| ***Mutation*** | ***FW primer*** | ***REV primer*** |
| --- | --- | --- |
|  |  |  |
| ***O8_EDED_PxxP*** | 5’-CAAGAAGATGAAGATCCAGTAAGACCTC-3’ | 5’-GGTCTTACTGGATCTTCATCTTCTTGGTGACTTTCTAGAAATGC-3’ |
| ***O8_EDED_VGF_AxxP*** | 5’-CAAGAAGATGAAGATGTAGGTTTTGCAGTAAGACCTC-3’ | 5’-GGTCTTACTGCAAAACCTACATCTTCATCTTCTTGGTGACTTTCTAGAAATGC-3’ |
| ***O8_EEEE_VGF_PxxP*** | 5’-CAAGAAGAAGAAGAAGTAGGTTTTCCAGTAAGACCTC-3’ | 5’-GGTCTTACTGGAAAACCTACTTCTTCTTCTTCTTGGTGACTTTCTAGAAATGC-3’ |
| ***O8_EDED_VGF_PxxP*** | 5’-CAAGAAGATGAAGATGTAGGTTTTCCAGTAAGACCTC-3’ | 5’-GGTCTTACTGGAAAACCTACATCTTCATCTTCTTGGTGACTTTCTAGAAATGC-3’ |
| ***O8_EDED_AAA_PxxP*** | 5’-CAAGAAGATGAAGATGCAGCAGCACCAGTAAGACCTC-3’ | 5’-GGTCTTACTTGCTGCTGCATCTTCATCTTCTTGGTGACTTTCTAGAAATGC-3’ |
| ***O8 FW + REV*** | 5’-TTTGGATCCTTTTTATAAATGGGAAACGCATTGAGAAAAC-3’ | 5’-AAAGAATTCAAAACGCGTTTATATATAGCGATAGGTGTCTTTGGAGAAGAGC-3’ |
|  |  |  |

**C)** Primers to generate VGF🡪AAA mutants ( indicated by ΔVGF) in B2, VI1422, NA-7 and NL4.3 Nef.

| ***Mutation*** | ***FW primer*** | ***REV primer*** |
| --- | --- | --- |
|  |  |  |
| ***B2_∆VGF*** | 5’-**CAAGAAGAGGATGAGGCAGCAGCACCAGTCAGACCTCAGG**-3’ | 5’-**GGTACCTGAGGTCTGACTGGTGCTGCTGCCTCATCC**-3’ |
| ***B2 FW + REV*** | 5’-TTTGGATCCTTTTTATAAATGGGTGGCAAGTTGTCAAAAAG-3’ | 5’-**TTTGAATTCTTTACGCGTTCATATATAGCGATAGGTGTCGCAGTCTTTGTAGTAC**-3’ |
| ***1422_∆VGF*** | 5’-**CAGGAGGAGGAAGAGGCAGCAGCACCAGTCAGACCCCAGG**-3’ | 5’-**GGTACCTGGGGTCTGACTGGTGCTGCTGCCTCTT**-3’ |
| ***1422 FW + REV*** | 5’-TTTTGGATCCATGGGGGGCAAATGGTCAAAATGTAG-3’ | 5’-**TTTGAATTCTTTACGCGTTCATATATAGCGATAGGTGTCGCAGTTTTTGTAGTACTCC**-3’ |
| ***NA-7/NL4.3∆VGF*** | 5’-**GAGGAGGAAGAGGCAGCAGCACCAGTCACACCTCAGGTACC**-3’ | 5’-**GGTCTTAAAGGTACCTGAGGTGTGACTGGTGCTGCTGCCTCC**-3’ |
| ***NL4.3/NA7 FW + REV*** | 5'-TTTGGATCCTTTTTATTAATTTATGGGTGGCAAGTGGTTCAAAACGTAGTGC-3’ | 5’-TTTGAATTCTTTACGCGTTCATATATAGGTGTCGCAGTTCTTGAAGTACTCC-3’ |
|  |  |  |

**D)** Primers for site specific mutagenesis of NA-7.GFP and SF2.GFP fusion proteins and NL4.3

| ***Mutation*** | ***FW primer*** | ***REV primer*** |
| --- | --- | --- |
|  |  |  |
| ***NA-7AxxA.GFP*** | 5’-GGAGGTGGGGTTTGCAGTCAGAGCTCAGGTACCTT-3’ | 5’-**AAGGTACCTGAGCTCTGACTGCAAACCCCACCTCC**-3’ |
| ***NA-7 VGF🡪AAA.GFP*** | 5’-CAAGAGGAGGAGGAGGCGGCGGCTCCAGTCAGACCTCAG -3’ | 5’-**CTGAGGTCTGACTGGAGCCGCCGCCTCCTCCTCCTCTTG**-3’ |
| ***NA-7 VGF🡪AGF.GFP*** | 3’-caagaggaggaggaggcggggtttccagtcagacctcag-5’ | 3’- ctgaggtctgactggaaaccccgcctcctcctcctcttg-5’ |
| ***NA-7 VGF🡪VAF.GFP*** | 3’- caagaggaggaggaggtggcgtttccagtcagacctcag-5’ | 3’- ctgaggtctgactggaaacgccgcctcctcctcctcttg-5’ |
| ***NA-7 VGF🡪VGA.GFP*** | 3’- caagaggaggaggaggtgggggctccagtcagacctcag-5’ | 3’- ctgaggtctgactggagcccccgcctcctcctcctcttg-5’ |
| ***SF2 VGF🡪AAA.GFP*** | 5’-CA**CAAGAGGAGGAAGAGGCGGCTGCTCCAGTCAGACCTCAGGT**-3’ | 5’-**ACCTGAGGTCTGACTGGAGCAGCCGCCTCTTCCTCCTCTTGTG**-3’ |
| ***SF2 VGF🡪AGF.GFP*** | 3’-cacaagaggaggaagaggcgggttttccagtcagacctcaggt-5’ | 3’- ctgaggtctgactggaaaccccgcctcttcctcctcttg-5’ |
| ***SF2 VGF🡪 VAF.GFP*** | 3’-cacaagaggaggaagaggtggcttttccagtcagacctcaggt-5’ | 3’-acctgaggtctgactggaaaagccgcctcttcctcctcttg-5’ |
| ***SF2 VGF🡪 VGA GFP*** | 3’-cacaagaggaggaagaggtgggtgctccagtcagacctcaggt-5’ | 3’-acctgaggtctgactggagcacccgcctcttcctcctcttg-5’ |
| ***NL4.3 VGF🡪 VGA*** | 3’-caagaggaggaagagg**c**gggttttccagtcagacctcag-5’ | 3’-acctgaggtctgactggaaacccc**g**cctcttcctcctcttg-5’ |
| ***NL4.3 VGF🡪 VAF*** | 3’- caagaggaggaagaggtgg**c**ttttccagtcagacctcag-5’ | 3’-acctgaggtctgactggaaaa**g**ccgcctcttcctcctcttg-5’ |
| ***NL4.3 VGF🡪 VGA*** | 3’- caagaggaggaagaggtgggt**gc**tccagtcagacctcag-5’ | 3’-acctgaggtctgactgga**gc**acccgcctcttcctcctcttg-5’ |
